# Simple, readily available clinical indices predict early and late mortality among patients with ANCA-associated vasculitis

**DOI:** 10.1186/s12882-017-0491-z

**Published:** 2017-02-23

**Authors:** Ágnes Haris, Kálmán Polner, József Arányi, Henrik Braunitzer, Ilona Kaszás, László Rosivall, Gábor Kökény, István Mucsi

**Affiliations:** 1 Nephrology Department, Szent Margit Hospital, 132 Bécsi út, Budapest, 1032 Hungary; 2 Pathology Department, Szent Margit Hospital, 132 Bécsi út, Budapest, 1032 Hungary; 3 Institute of Pathophysiology, Semmelweis University, 4 Nagyvárad tér, Budapest, 1089 Hungary; 4 Department of Medicine (Nephrology), University of Toronto, Kidney Transplant Program, Toronto General Hospital, University Health Network, 585 University Avenue, Toronto, M5G 2 N2 ON Canada

**Keywords:** ANCA, BVAS, Comorbidity, Immunosuppression, Outcome, Vasculitis

## Abstract

**Background:**

The early identification of patients with ANCA-associated vasculitis (AAV) who are at increased risk for inferior clinical outcome at the time of diagnosis might help to optimize the immunosuppressive therapy. In this study we wanted to determine the predictive value of simple clinical characteristics, which may be applicable for early risk-stratification of patients with AAV.

**Methods:**

We retrospectively analyzed the outcome of 101 consecutive patients with AAV receiving a protocolized immunosuppressive therapy. Baseline Birmingham Vasculitis Activity Score (BVAS) and non-vasculitic comorbidities were computed, then predictors of early (<90 days) and late (>90 days) mortality, infectious death, relapse and end stage kidney disease (ESKD) were evaluated.

**Results:**

The baseline comorbidity score independently predicted early mortality (HR 1.622, CI 1.006–2.614), and showed association with infectious mortality (HR 2.056, CI 1.247–3.392). Patients with BVAS at or above median (=21) had worse early mortality in univariable analysis (HR 3.57, CI 1.039–12.243) (*p* = 0.031), and had more frequent relapses (*p* = 0.01) compared to patients with BVAS below median.

**Conclusions:**

Assessing baseline comorbidities, beside clinical indices characterizing the severity and extension of AAV, might help clinicians in risk-stratification of patients. Future prospective studies are needed to investigate whether therapies based on risk-stratification could improve both short term and long term survival.

## Background

The outcome of ANCA-associated vasculitis (AAV) has improved significantly since the introduction of immunosuppressive therapy. On the other hand, both the disease and the cytotoxic treatment are associated with considerable morbidity and mortality [1, 2]. Ideally, patients should receive a treatment specifically tailored to the severity of their disease. Other factors, however, such as age, the extent of organ involvement and also baseline comorbidities may influence the outcome [3–7]. Therefore, in order to optimize the intensity of immunosuppression and to optimize outcomes, these factors would be important to consider when planning the treatment schedule of the individual patient at the time of diagnosis.

The Birmingham Vasculitis Activity Score (BVAS) is a reliable tool to estimate the severity and extent of the disease [8]. However, studies investigating it’s predictive value on survival reported conflicting results [9–13]. Beside the activity of AAV, patients may have comorbid conditions, that may also have an impact on their survival. Comorbidity scores are useful clinical tools for risk-stratification of patients with chronic disorders, and the role of comorbid conditions has been emphasized in the mortality of dialysis patients [14, 15]. Therefore, it seems reasonable to include comorbidity assessment in the initial risk-startification at the time of presentation with AAV.

The aim of our study was to determine if simple clinical and laboratory characteristics, readily available at the time of diagnosis would predict mortality in patients with BVAS. We assessed BVAS, and utilized a simplified score by computing the most important baseline non-vasculitic comorbidities for risk-stratification of patients with AAV.

## Methods

All consecutive patients, diagnosed with AAV at our nephrology center between January, 1998 and June, 2013 were considered for this study. One patient, who died within the first month, and 3, who were lost to follow-up were excluded. Last follow-up was the date of death or the end of study (December 31, 2013). Patients who survived longer than the 8 years of follow-up (*n* = 15) were censored at that time.

The diagnosis of necrotizing small vessel vasculitis was defined according to the criteria of Chapel Hill consensus conference [16, 17], by clinical presentation compatible with AAV, positive ANCA serology and/or kidney biopsy. Histological result confirmed the presence of crescentic/necrotizing glomerulonephritis in all but 10 subjects, in whom renal biopsy was not performed either because of life threatening condition or due to refusal by the patients. All these 10 patients were ANCA positive. Estimated GFR (eGFR) was computed with CKD-EPI equation [18], and BVAS (version 3) was calculated by scoring symptoms in 9 organ systems (general, cutaneous, mucus membranes/eyes, ENT, chest, cardiovascular, abdominal, renal, and nervous system) at admission [8] (Evaluelogix software by EPS Research Ltd). Baseline comorbidity score was assessed by determining conditions that had been present before the AAV, namely history of myocardial infarction, congestive heart failure, peripheral vascular disease, cerebrovascular disease, chronic pulmonary disease, peptic ulcer disease, liver disease, diabetes or malignancy. Scores were given 0 if no comorbidity, 1 if a single comorbidity, 2 if two or more comorbidities existed.

Patients received protocolized therapy during the entire observational period: 500–1000 mg intravenous (iv) metlyprednisolone (MP) for three consecutive days, followed by 1 mg/kg/day per os for one month, then daily 48 mg in the second, 36 mg in the third, 24 mg in the fourth, 16 then 12 mg in the fifth, and 8 then 4 mg in the sixth months, continued with the maintenance dose of 4 mg/day, and 10 mg/kg iv bolus cyclophosphamide monthly for six months, repeated at months 9 and 12. For subjects older than 65 years the dose of immunosuppressive medications was decreased by 15%, and for older than 70 years by 20%, but the CYC dose was not modified by the glomerular filtration rate. In 92 patients five plasmapheresis sessions were also performed. Eighty-six patients followed the protocol strictly. When we analyzed their data separately, the results were comparable to the findings in the whole cohort. After twelve months azathioprine was introduced, accompanied by 4 mg methylprednisolone given daily or every other day as long-term maintenance therapy, at the discretion of the attending nephrologist.

In case of relapse, the induction immunosuppressive regime was repeated.

Remission was defined as disappearance of clinical disease activity and stabilization or improvement of the kidney function. Resolution of hematuria was also criteria for remission, but persistent proteinuria was considered as the consequence of glomerular damage. In patients who remained dialysis dependent we considered remission if the extrarenal manifestations and the hematuria completely ceased. Relapse was defined as recurrence of presenting symptoms or appearance of a new organ involvement attributable to AAV. Those, in whom remission could not be achieved, who died due to active vasculitis, or had low grade of persistent “grumbling disease” were considered as treatment resistant patients.

The main exposure variables were the comorbidity score (the sum of comorbidities at the time of admission) and the BVAS score (categorized as below or above median [median = 21] score).

The primary end points were all cause early (<90 days) and late (>90 days) mortality. Secondary end points consisted of deaths due to infections, rate of relapse and end stage kidney disease (ESKD).

Statistical analyses were performed using SPSS 20.0 (IBM, Chicago, IL) and STATA MP version 12 (Stata Corporation, College Station, TX). Variables were reported as mean (SD) or median and range, comparison between groups was analyzed by Student’s t-tests, Mann–Whitney U tests or *χ* ^2^ tests, as appropriate. Mortality risk was calculated by Kaplan-Meier method, and log-rank tests to compare groups. Predictors of death were evaluated separately for early (<90 days) and late (>90 days) mortality.

Patients with BVAS below and at or above median were compared. Although the relatively small number of events limited multivariable analyses [19], for this purpose those variables were selected, that were considered important predictors of outcomes of AAV based on clinical experience or the results of the univariable analyses.

Multivariable models were sequentially adjusted for age, serum albumin, HD dependency on admission, and ANCA type (negative, p- or c-ANCA). Serum CRP was not used in the multivariable models due to the small number of events and also because of its strong correlation with serum albumin.

Logistic regression models were used to analyze the association between exposure variables and relapse, since we considered all relapses for these analyses and we did not consider the time to events.

Results are expressed as hazard ratios (HRs) with 95% confidence intervals (CIs) and p values. All tests were two-tailed, unadjusted for multiple comparisons, and *p* values of < 0.05 were considered significant.

## Results

Baseline data of the 101 individuals are presented in Table 1. Subjects with BVAS at or above median (median BVAS = 21) had lower Hgb (*p* = 0.017), more c-ANCA positivity (*p* = 0.024), and needed HD on admission more often (*p* = 0.012), compared to the individuals with BVAS below median.

**Table 1:** Demographics, baseline data and comorbidities at time of diagnosis (mean (SD) or median and range)

Variable	All patients	Patients with BVAS ≥ 21	Patients with BVAS < 21	*p* value
*n* of patients	101	63	38	
Age (years)	61.4 (13)	60.2 (14)	63.3 (11)	0.237
Male/female	40/61	27/36	13/25	0.389
Time from first symptoms to diagnosis (months)	5.0 (1–36)	5.0 (1–24)	6.0 (1–36)	0.822
Hemoglobin (g%)	8.4 (1.4)	8.1 (1.3)	8.8 (1.5)	*0.017*
Erythrocyte sedimentation rate (mm/h)	98 (5–138)	98 (5–138)	99 (14–136)	0.762
Serum albumin (g/l)	31.3 (5.4)	30.8 (5.3)	32.0 (5.6)	0.285
CRP (mg/l)	29 (1–221)	40 (2–221)	24 (1–152)	0.054
Urinary protein excretion (mg/day)	1456 (38–8474)	1259 (38–8474)	1843 (184–7344)	0.143
Serum creatinine (umol/l)	554 (84–1904)	573 (146–1904)	428 (84–1722)	0.060
HD requirement on admission (*n*, %)	56 (55%)	41 (65%)	15 (40%)	*0.012*
BVAS	21 (11–34)	24 (21–34)	15 (11–20)	*<0.001*
p-/c-ANCA positivity (*n*)*	57/36	33/29	24/7	*0.024*
Anti-MPO level in p-ANCA positive patients (IU/ml)	67 (6–100)	67 (6–100)	70 (11–100)	0.807
Anti-PR3 level in c-ANCA positive patients (IU/ml)	100 (32–100)	100 (50–100)	82 (32–100)	0.354
Dose of iv pulse MP** mg/kg/bw	11.7 (4.1)	12.7 (4.2)	10.1 (3.3)	*0.002*
Dose of iv bolus CYC*** mg/kg/bw	9.7 (1.6)	9.8 (1.6)	9.5 (1.6)	0.371
Cumulative dose of MP (mg)	11640 (3006–32334)	11238 (3006–32334)	12332 (5076–26364)	0.621
Follow-up time (days)	963 (30–3000)	843 (30–3000)	1393 (51–3000)	0.231
Organ involvement n (%)				
Renal	101 (100)	63 (100)	38 (100)	1.000
Respiratory tract	43 (43)	38 (60)	5 (13)	*<0.001*
Ear-throat-nose	39 (39)	33 (52)	6 (16)	*<0.001*
Musculoskeletal	55 (55)	33 (52)	22 (58)	0.590
Skin	16 (16)	9 (14)	7 (18)	0.581
Eyes	5 (5)	4 (6)	1 (3)	0.648
Gastrointestinal	9 (9)	9 (14)	0 (0)	*0.013*
Nervous system	17 (17)	14 (22)	3 (8)	0.062
Cardiovascular	5 (5)	5 (8)	0 (0)	0.154
Baseline comorbidities n (%)	963 (30–3000)	843 (30–3000)	1393 (51–3000)	0.231
History of:				
Coronary artery disease	11 (11)	9 (14)	2 (5)	0.201
Congestive heart failure	5 (5)	4 (6)	1 (3)	0.648
Peripheral vascular dis.	1 (1)	1 (2)	0 (0)	1.000
Cerebrovascular disease	11 (11)	7 (11)	4 (11)	1.000
Chronic pulmonary dis.	16 (16)	9 (14)	7 (18)	0.581
Peptic ulcer disease	8 (8)	6 (10)	2 (5)	0.707
Liver disease	5 (5)	4 (6)	1 (3)	0.648
Diabetes mellitus	8 (8)	3 (5)	5 (13)	0.149
Malignancy	7 (7)	3 (5)	4 (11)	0.421
Number of patients with				
0	50	30	20	0.862
1	30	19	11	
2 or more comorbidity scores	21	14	7	

*Eight patients were ANCA negative, all of them had renal biopsy which proved the diagnosis of pauci-immune crescentic glomerulonephritis

**MP – methylprednisolone, administered for 98 patients

***CYC – cyclophosphamide, administered for 95 patients

Comorbidity scores were given 0 if no comorbidity, 1 if a single comorbidity, 2 if two or more comorbidities existed

Treatment protocol was strictly followed with only few exceptions, as excluded iv MP pulses in 1 and 2 patients and excluded CYC boluses in 3 and 1 patients in the BVAS at or above and below median groups, respectively; CYC was administered orally in 2 patients in the BVAS below median group. Subjects with BVAS at or above median got higher dose of pulse MP (*p* = 0.002) compared to the individuals with BVAS below median, but the dose of CYC and the cumulative dose of MP did not differ between the groups (Table 1).

**Fig. 1:**
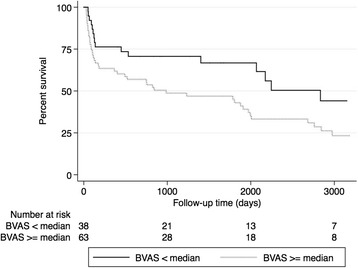
Kaplan-Meier survival curves of patients with BVAS at or above and below median in the entire observation period (*p* = 0.028, log-rank test)

The median survival in the study sample was 1877 (95%CI 753–2246) days. Mortality during the first year was 33%. Nineteen patients died within the first 90 days (“early mortality”), and 41 after the 90^th^ day of follow-up (“late mortality”). The cumulative probability of survival was 0.441 (95%CI 0.231–0.633) versus 0.233 (95%CI 0.126–0.359) (*p* = 0.028) in patients with a BVAS score below versus at or above median, respectively (Fig. 1). The cumulative probability of early (within 90 days after diagnosis) survival was also worse in patients with higher BVAS: 0.921 (95%CI 0.775–0.974) versus 0.746 (95%CI 0.619–0.836) (*p* = 0.031). Early mortality was also predicted by baseline comorbidity score, albumin, CRP and HD requirement on admission in urivariable Cox regression analysis (Table 2). In a multivariable model adjusted for BVAS, age, serum albumin, ANCA type and HD requirement on admission comorbidity score remained a significant predictor for early mortality (HR 1.622, CI 1.006–2.614, *p* = 0.047) (Table 3).

**Table 2:** Predictors of “early mortality” in univariable Cox regression analysis

	HR	95% CI	*P* value
Age	1.035	0.994–1.078	0.097
Albumin	0.891	0.818–0.970	*0.007*
ANCA type – ANCA neg	REF	REF	REF
pANCA	0.604	0.215–1.695	0.338
cANCA	0.516	0.067–3.945	0.524
BVAS median	3.57	1.039–12.243	*0.043*
CRP	1.011	1.003–1.018	*0.005*
Comorbidity score	1.707	1.176–2.477	*0.005*
HD on admission	3.404	1.130–20.260	*0.030*

**Table 3:** Comorbidity score predicts “early mortality” in multivariable Cox regression analysis. Table shows the parameters of the “comorbidity score” variable in different models

	HR	95% CI	*P* value
Model 1	1.707	1.176–2.477	*0.005*
Model 2	1.752	1.225–2.506	*0.002*
Model 3	1.694	1.072–2.677	*0.024*
Model 4	1.622	1.006–2.614	*0.047*

Model 1: comorbidity score

Model 2: Model 1 + BVAS median

Model 3: Model 2 + Age, serum albumin

Model 4: Model 3 + HD dependency on admission and ANCA type (c-versus p-ANCA)

Late (>90 days after diagnosis) mortality was predicted by age, comorbidity score and HD requirement on admission in univariable analysis (Table 4). In the most fully adjusted model adjusted for age, serum albumin, HD dependency on admission and ANCA type, comorbidity was not a significant predictor any more. In this model, however, BVAS independently predicted all cause late mortality (HR 2.408, 95%CI 1.081–5.362, Table 5).

**Table 4:** Predictors of “late mortality” in univariable Cox regression analysis

	HR	95% CI	*P* value
Age	1.059	1.028–1.092	*<0.001*
Albumin	0.950	0.895–1.009	0.094
ANCA type – ANCA neg	REF	REF	REF
pANCA	0.658	0.341–1.268	0.211
cANCA	0.674	0.159–2.853	0.592
BVAS median	1.483	0.768–2.865	0.241
CRP	0.999	0.993–1.007	0.993
Comorbidity score	1.526	1.106–2.106	*0.010*
HD on admission	2.157	1.131–4.116	*0.020*

**Table 5:** BVAS predicts “late mortality” in multivariable Cox regression analysis. The table shows the parameters of the “BVAS median” variable in the different models

	HR	95% CI	*P* value
Model 1	1.483	0.768–2.865	0.241
Model 2	2.073	1.030–2.435	*0.041*
Model 3	2.558	1.251–5.231	*0.010*
Model 4	2.408	1.081–5.362	*0.031*

Model 1: BVAS median

Model 2: Model 1 + comorbidity score

Model 3: Model 2 + Age, serum albumin

Model 4: Model 3 + HD dependency on admission and ANCA type (c- versus p-ANCA)

Seven patients died of infections (37%), 4 of cardiovascular diseases (21%) and 7 of AAV activity (37%) within the first 90 days. Late mortality occurred from infections in 13 (32%), cardiovascular diseases in 12 (29%), active AAV during relapse in 6 patients (15%). Reason for death was unknown in 10 additional cases (1 early, 9 late mortality), and late malignancy was responsible for one death. The following types of infections were documented: bacterial and fungal respiratory tract infections, pulmonary abscess, cerebral abscess, sepsis, disseminated herpes zoster. Both comorbidity and BVAS predicted infectious mortality (HR 2.191, 95%CI 1.486–3.231; HR 3.792, 95%CI 1.111–12.949, respectively) in univariable models. The predictive value of comorbidity and BVAS remained significant after adjustment for age, serum albumin, ANCA types, and HD dependency (HR 2.056, 95%CI 1.247–3.392; HR 5.079, 95%CI 1.396–18.480, respectively).

By induction immunosuppression remission was achieved in all but one patient, who survived more than 90 days (81 patients, 80%). On the long term, ESKD developed in 3 patients who had not required dialysis at diagnosis, but suffered renal failure likely due to low grade persistent disease activity. Thirty-seven patients remained dialysis dependent at study end. Serum creatinine and eGFR in patients who were off dialysis at the end of follow-up (*n* = 64) were 168 umol/l (83–434) and 33 ml/min (11–88), respectively. In those, who had BVAS at or above median on admission, serum creatinine at the end of follow-up was significantly higher (191 umol/l (88–418)), compared to patients with BVAS below median (143 umol/l (83–434), *p* = 0.041). The corresponding eGFR values were 26 and 38 ml/min (11–75 and 12–88, *p* = 0.092, respectively). Frequency of long term HD dependency in patients with BVAS at or above and below median did not differ significantly.

Forty relapses occurred in 24 patients, 10 of them experienced 2–4 relapses. The proportion of patients with relapses was 30% in the BVAS at or above median and 13% in the BVAS below median group (*p* = 0.052). There was significant difference in the number of relapses between the subgroups with BVAS at or above and below median (34 relapses in 63 patients vs. 6 relapses in 38 patients, *p* = 0.01). Although BVAS showed association with relapse in univariable logistic regression model (OR = 1.130 CI 1.028–1.243), after correcting for the type of ANCA (c-ANCA versus p-ANCA), BVAS was not a significant predictor of relapse any more.

## Discussion

The main result of our analysis is that in AAV patients with predominant renal and pulmonary involvement, comorbidity score independently predicted short term survival. It also proved to be a predictor of infectious mortality. On protocolized immunosuppressive therapy, patients, who had high BVAS at baseline, had significantly poorer short term survival and more frequent relapses than subjects with lower than median score. When analyzing early and late mortality separately, BVAS did not predict outcome in univariable analysis.

BVAS, originally designed to standardize disease assessment in AAV, shows good correlation with clinical activity of the disease [8]. Flossmann et al. documented, that BVAS was a significant predictor of mortality by analyzing the data of patients recruited for randomized controlled trials. Patients in that study were somewhat different from the ones enrolled in ours, since the median BVAS was lower, renal function was less severely compromised, and subjects with life-threatening pulmonary hemorrhage were excluded [1]. On the contrary, predictive value of BVAS was not found in several other investigations. Bakoush and coworkers followed 83 patients; neither survival nor ESKD was predicted by BVAS in their cohort, with less severe renal failure compared to our patients [9]. In Japanese patients with MPO-ANCA disease, no association was found between BVAS and mortality during the two years follow-up [20]. In another investigation there was no difference between the baseline BVAS of survivors and non-survivors; baseline BVAS did not, but BVAS at 1 and 3 months predicted survival [21].

The difference in the association between BVAS and outcome in these cohorts and ours can be due to a variety of factors. Event number, therefore statistical power, patient selection, disease severity and treatment approach were quite heterogeneous across these studies. It also seems important to differentiate early and late survival, as the hazard of mortality is not proportional in these periods. We have defined the timeframe of early death in 3 months, as risk of severe complications of AAV, also intensity of immunosuppression are the highest during this period.

To our knowledge, only one study has investigated the association between comorbidities and risk of all cause death in AAV patients. Little et al. found, that the Karnofsky performance score, but not the non-vasculitic comorbidity showed independent association with mortality [22]. Although we did not include Karnofsky performance in our dataset, we found a significant association of comorbidity and early mortality, and this relationship remained independent of other important clinical characteristics.

We did not find other investigations assessing the association between comorbidities and infectious death. Importantly, this reveals the complexity of treatment of AAV patients: likely those without any comorbidity may tolerate aggressive immunosuppression and AAV better, compared to subjects suffering from various chronic disorders. Remarkably, in another study, accumulation of adverse events in the first year of treatment - which influenced survival significantly – was independently associated with age and renal impairment [2]. Based on these latter findings we propose, that not only age and kidney function, but also the presence of non-vasculitic comorbidities present high risk status for adverse events, especially for infections, which may provide an explanation for the increased mortality.

Our findings are in accord with several other reports showing that the severity of kidney disease at baseline is an indicator of poor prognosis [1, 6, 9, 10]. Similarly, high levels of the inflammatory markers (CRP, albumin, etc.) confer an increased early mortality risk for the individual patient [5, 7]. The applicability of these predictors is important, as these are readily available at the first presentation of the patient.

The frequency of ESKD did not differ in the BVAS groups in our cohort, similarly to other investigations [4, 23]. We found more frequent relapses in patients with higher BVAS; BVAS predicted relapse in Cox regression analysis, but the association was not significant after adjustment for ANCA type. The likely explanation for this observation is, that the higher BVAS in our cohort, comprising patients with both respiratory tract and kidney involvement, was associated with c-ANCA disease, which characteristically confers a higher risk of relapse compared to *p*-ANCA positive vasculitis [24]. In comparison, in a study investigating patients exclusively with c-ANCA positivity, relapses occurred more often in those who presented with lower BVAS, compared to more severe cases [25]. A possible explanation for this difference can be the different case mix: cohorts with predominantly upper respiratory tract involvement but no kidney disease have lower BVAS but more relapses than those with renal AAV [26].

Our study has several limitations. Most importantly, BVAS was calculated retrospectively. Nevertheless, detailed source data provided reliable information, and the strictly followed treatment protocol also assisted our analysis. Mortality rate was fairly high, which likely can be explained by very late referrals. This resulted in advanced renal failure and extensive manifestations of AAV in most of our patients. The extensive comorbidities might also have contributed to the observed high mortality.

In conclusion, baseline comorbidities influence both short and long term outcome of patients with AAV. Risk-stratification would help clinicians to tailor therapy individually, which might further improve the outcome. Future prospective treatment studies are needed to assess whether scoring systems based on comorbidities and BVAS help to individualize therapies in order to improve short and long term survival.

## Abbreviations

AAV: ANCA-associated vasculitis
BVAS: Birmingham Vasculitis Activity Score
CI: Confidence interval
CYC: Cyclophosphamide
eGFR: Estimated GFR
ESKD: End stage kidney disease
HR: Hazard ratio
iv: Intravenous
MP: Methylprednisolone
OR: Odds ratio
